# Logical Attacks and Countermeasures for Fingerprint On-Card-Comparison Systems †

**DOI:** 10.3390/s20185410

**Published:** 2020-09-21

**Authors:** Benoit Vibert, Jean-Marie Le Bars, Christophe Charrier, Christophe Rosenberger

**Affiliations:** Ensicaen, Normandie University, Unicaen, CNRS, GREYC, 14000 Caen, France; benoit.vibert@ensicaen.fr (B.V.); jean-marie.lebars@unicaen.fr (J.-M.L.B.); christophe.charrier@unicaen.fr (C.C.)

**Keywords:** fingerprint classification, logical attack, evaluation, robustness, fingerprint features

## Abstract

Digital fingerprints are being used more and more to secure applications for logical and physical access control. In order to guarantee security and privacy trends, a biometric system is often implemented on a secure element to store the biometric reference template and for the matching with a probe template (on-card-comparison). In order to assess the performance and robustness against attacks of these systems, it is necessary to better understand which information could help an attacker successfully impersonate a legitimate user. The first part of the paper details a new attack based on the use of a priori information (such as the fingerprint classification, sensor type, image resolution or number of minutiae in the biometric reference) that could be exploited by an attacker. In the second part, a new countermeasure against brute force and zero effort attacks based on fingerprint classification given a minutiae template is proposed. These two contributions show how fingerprint classification could have an impact for attacks and countermeasures in embedded biometric systems. Experiments show interesting results on significant fingerprint datasets.

## 1. Introduction

Biometrics is a commonly used technology for unlocking smartphones, secure border controls or physical access to buildings. Yet, biometrics data are sensitive, since it is not possible in general to revoke them in case of an attack. Thus, these data have to be protected as well as possible. In the case of digital fingerprints, the reference template (a set of minutiae) is usually stored in a secure element (SE) (such as e-passports). Due to the limitation of memory size and computational capabilities, the reference template is stored following the ISO Compact Card II standard [1]. This representation facilitates the comparison between the reference template and the probe sample. The security of embedded biometric systems on a SE is therefore a primary requirement.

**Figure 1:**
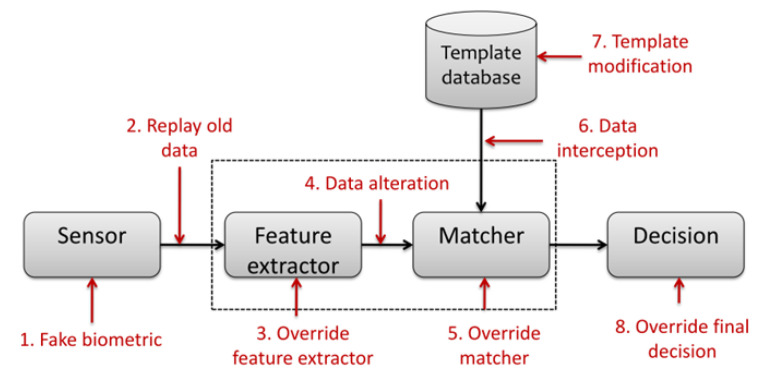
Locating vulnerabilities on a biometric system (defined by [2]).

Regarding security, biometric systems have many vulnerabilities. As presented by Ratha et al. [2] and more recently Jain et al. [3], authors have classified the attacks of a generic biometric system into eight categories (as summarized in Figure 1). For each of the identified points, there are different types of attacks. Uludag and Jain [4], Martinez [5] and Soutar [6] considered points 2 and 4 to perform a hill-climbing attack. This attack can be performed by an application that continuously sends random data to the system. The application retrieves the matching score between the reference template and the probe sample and continues its disturbances only when the correspondence score increases and until the acceptance threshold is reached. Note that on-card-comparison (OCC) systems never provides as output the matching score in order to avoid this attack, and the decision is realized inside the secure element.

In general, the attacker has to generate a biometric template to carry out an attack. Considering embedded biometric comparison algorithms on a SE, the attacker sends random probes in an attempt to pretend to be the legitimate user until success. Since theoretically and in the worst case, the attacker generates all possible combinations of template, this attack is called brute force. Different studies have been investigated to prevent this type of attack [4,5]. Another attack consists in using a biometric probe calculated from impostor’s own biometric data, this attack is called “zero effort”. This attack has very little chance of being efficient, but it can serve as a basis for more advanced ones.

In this work, we consider the vulnerability on point 4 (namely data alteration in Figure 1) within the context of an OCC implementation (the most secure case). It corresponds to the scenario where an impostor tries to impersonate a legitimate user in order to open its smartphone protected by a fingerprint sensor as for example by injecting digital attempts. To the best of our best knowledge, very few study on the a priori information that could be exploited by an attacker has been carried out in the literature [7].

We summarize in the following the main contributions of the paper. The first one concerns the identification of useful information for an attacker to impersonate a legitimate user identity while using a biometric system. Very few works in the literature have investigated this question. We believe it is important for the security of biometric systems. We investigated four kinds of information that could be obtained by an attacker: (1) the type of sensor, (2) the fingerprint image resolution, (3) the minutiae number and (4) the fingerprint type. This methodology is new and could be used for any other a priori information. The second contribution is the design of a fingerprint classification method only considering information contained in a minutiae template (i.e., without any access to the original image). Once again, very few works have concerned this topic in the literature. This method could be useful to detect brute force and zero effort attacks by checking the fingerprint type of probes. The common point of these two contributions concerns the fingerprint classification that could be useful both for attackers and defenders of biometric systems.

The paper is organized as follows. Section 2 focuses on the impact of a priori knowledge an impostor could use for attacks. In Section 3, we address the problem of fingerprint classification from a minutiae template in order to detect brute force and zero effort attacks. Finally, we conclude and give some perspectives of this study in Section 4.

## 2. Which a Priori Information Could Be Useful for an Impostor?

**Figure 2:**
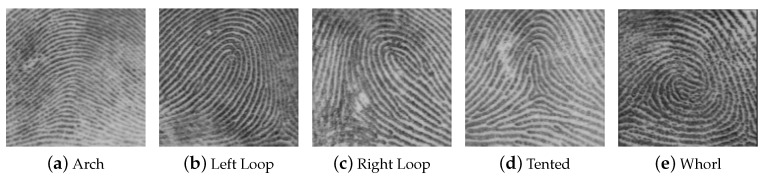
The five types of fingerprints defined by Henry (source [7]).

Our hypothesis is that an attacker has a logical access to the system and has the possibility to send fake biometric templates to the OCC by exploiting some a priori information. The available knowledge on any biometric sensor is categorized as:

- The fingerprint class, according to the Henry’s classification [8] for which five classes were identified: Arch, Left Buckle, Right Buckle, Tent and Spiral [9,10], as illustrated in Figure 2.
- Sensor type used during the enrollment (among capacitive and optical);
- Image resolution;
- Number of extracted minutiae in the reference template.

In this section, we propose to investigate their impact on the success of attacks. We assume that an attacker can only send digital templates or images to the OCC, thus implementing the vulnerabilities 2 and 4 defined in the Ratha model by (1) modifying the resolution of the image supplied at the sensor output thereby influencing the minutiae extraction, (2) setting the fingerprint class, (3) providing information on the type of sensor used during the enrollment process and (4) when the attack is performed right after the minutiae extraction process, the attacker can know the number of minutiae extracted saved in the reference template in the SE. All extracted minutiae are stored in a template following the ISO Compact Card II format.

We want to quantify the contribution of the attacker’s knowledge of the parameters used by the sensor to increase the efficiency of an attack. This probability is based on the False Acceptance Rate (FAR) which can be considered as the probability of a successful attack. We define $b_{z}$ as the biometric reference template for the user *z* and *D* a comparison algorithm based on a distance between a reference and a biometric probe. The success of an attack by an impostor is given by:

(1) $$\begin{array}{c} FAR_{A}\left(\epsilon \right)=P[D\left(b_{z},A_{z}\right)\leq \epsilon ], \end{array}$$

where $FAR_{A}$ is the probability of a successful attack for a decision threshold ϵ. The biometric probe sample $A_{z}$ is generated by the impostor taking into account all the information he/she knows about the user *z* or the biometric system. Our goal is to estimate the advantage for an attacker to build $A_{z}$ when he/she knows the fingerprint class $C_{z}$, the sensor type $S_{z}$, the number of minutiae $MN_{z}$, or the resolution of the fingerprint image $R_{z}$ used for generating the reference template of the user *z*. In the next section, we design the experimental protocol in order to estimate the advantage an impostor has knowing some or all information.

### 2.1. Experimental Protocol

We have to define many aspects of the experimental protocol such as the biometric datasets, the matching algorithms, the testing scenarios and the software platform for running experiments.

#### 2.1.1. Biometric Databases

**Figure 3:**
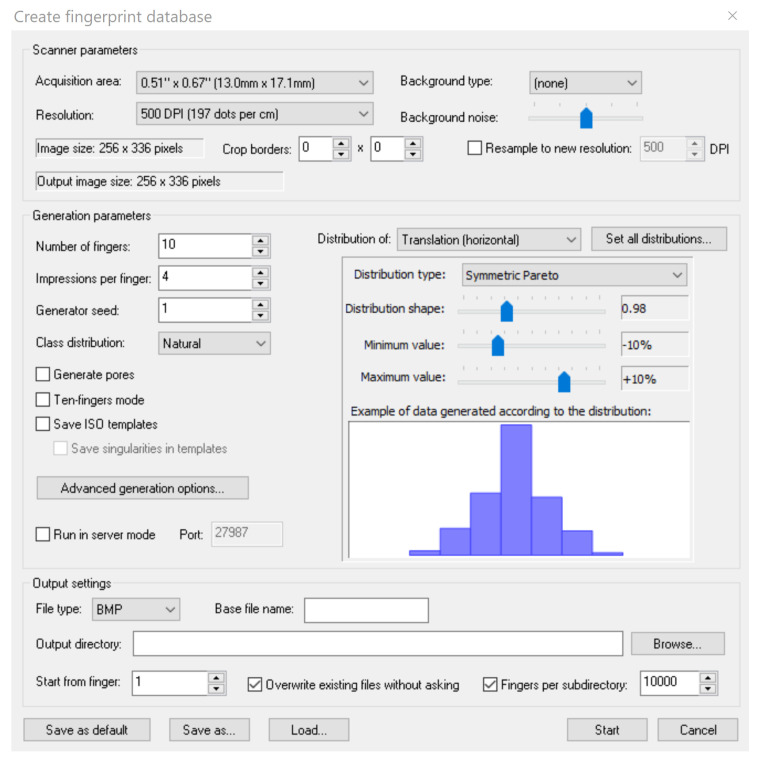
Sfinge: a software for the generation of synthetic fingerprints.

We used the SFinge software [11] to generate different synthetic biometric databases (Figure 3). This software tool is well known in biometrics as it has already been used during Fingerprint Verification Competitions (FVC). Previous works [12,13] demonstrated that SFinge produces synthetic fingerprints with similar behaviors in terms of recognition rates to those obtained from real databases. During the generation of synthetic fingerprints, the software allows us to select many a priori information (type of sensor, number of minutiae, image resolution and especially the fingerprint class).

For each of the four a priori information, two types of databases are designed:

1. **Reference database**: this database contains the reference templates of all users. We randomly generated one sample per user for 500 individuals (given a random and distinct seed for each user). This database contains 500 fingerprints;
2. **Attack database**: we generated a database with 1000 different fingerprint samples (one sample per user). This database has been randomly generated (by using different seeds than the reference database) and is used for attacks.

When using SFinge, we can choose the type of sensor among capacitive and optical. This leads to the construction of four databases (a reference database and an attack one by sensor type). Considering the resolution level of the fingerprint image, we have three possible values (250 dpi, 500 dpi, 1000 dpi) inducing six databases. For the number of minutiae, we have created two categories (number of minutiae <38 or >38) inducing four databases. Finally, when considering the fingerprint class (Arch, Left Loop, Right Loop, Tented and Whorl), 10 databases are generated (two per class).

In order to set the decision threshold ϵ used in Equation (1), we propose to use the threshold value when the system is defined at the Equal Error Rate value (EER). It is an arbitrary choice, as this operating point is always accessible for any matching algorithm. In order to compute this EER value for a given matching algorithm, we generated a dedicated database using SFinge with the default parameters, that we call DB_SFinge. The only parameters we have set are the number of users (100) and the number of templates per user (8). Finally, we get a total of 800 fingerprints.

#### 2.1.2. Matching Algorithms

In this study, we used two matching algorithms from the research community in biometrics:

- Bozorth3 algorithm [14]: The EER value of this algorithm was calculated using the DB_SFinge database. The value obtained was equal to 1.03% with a decision threshold value ϵ= 26.8;
- Minutia Cylinder-Code (MCC) algorithm [15]: The EER value of this algorithm was also computed using the DB_SFinge database. The value obtained was equal to 0% for a decision threshold ϵ=0.0315.

#### 2.1.3. Testing Scenarios

For any attack, an impostor provides a biometric probe to be authenticated as a legitimate user. Two scenarios are used to simulate an attack:

1. Scenario 1: we simulated a brute force attack. We randomly selected 500 min templates, following a uniform distribution, in the database generated using SFinge, which constitutes the reference database. The attack database was generated by building 1000 biometric templates randomly but respecting the ISO format, itself coming from SFinge.
2. Scenario 2: For each of the given a priori information, a reference database was generated with the SFinge software containing 500 min templates. In addition, for each of the a priori information, an attack database containing 1000 biometric probe templates is generated and is compared with the reference database. For example, considering the sensor type, we obtain four comparisons as shown in Table 1.

#### 2.1.4. Implementation within the Evabio Platform

**Figure 4:**
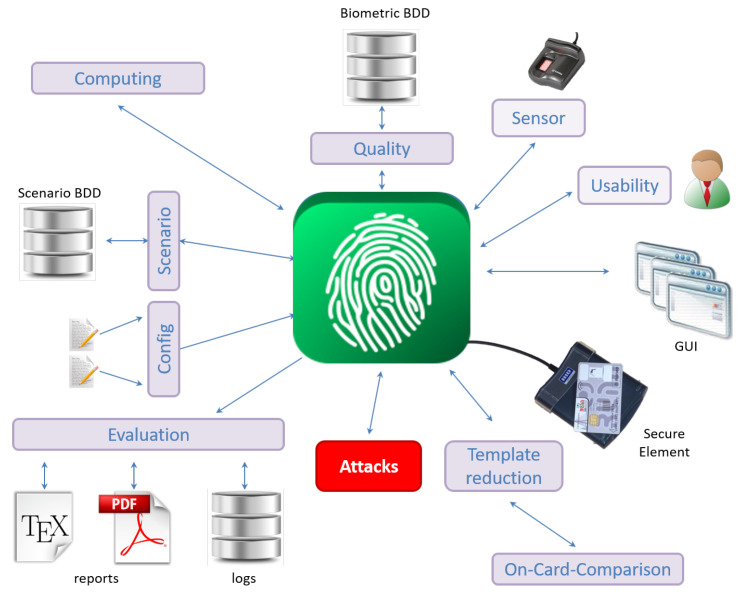
General diagram of the EvaBio platform (defined in [16]).

In order to evaluate the impact of an a priori information on the efficiency of an attack, we use the EVABIO platform [16] to characterize its influence on the matching decision. The EVABIO platform has been designed in our research lab in order to facilitate the evaluation of biometric systems with different modules (see Figure 4). In previous studies, we showed the benefit of this platform to speedup the computation time of the performance evaluation of biometric systems [17] or assessing their security [18] thanks to different modules.

We have developed a new attack module to carry out this study in order to test different attack methods when evaluating an OCC fingerprint system. In this study, the Attacks module was updated because it contains methods for testing the useful knowledge for an attacker such as the sensor type, the image resolution captured by the sensor, the fingerprint class or the number of minutiae extracted from the image. Given each information, we determined whether this type of knowledge is important for an attacker to succeed in impersonating individuals. This module also contains a method for generating an ISO compliant biometric template using SFinge. It is possible to generate random fingerprint templates to attack the matching algorithms. The Evaluation module generates performance metrics such as $FAR_{A}$ for different values of the decision threshold ϵ.

### 2.2. Experimental Results

In this section, we present the results of this experimental study for each separately considered a priori information.

#### 2.2.1. Sensor Type

Regarding the knowledge of the sensor type used to generate the biometric reference of the individual, we compute the value $FAR_{A}$ for the two scenarios described above when we set the value of the decision threshold with respect to the used comparison algorithm, as described in Section 2.1.2. Table 2 gives the probability value of a successful attack $FAR_{A}$ for each sensor type and the two comparison algorithms. We can clearly conclude that the knowledge of the sensor type used during enrollment does not help the attacker.

#### 2.2.2. Number of Extracted Minutiae

With the knowledge of this a priori information, (the number of minutiae in the biometric reference of the individual), we compute the $FAR_{A}$ value for the two scenarios described above when we set the value of the decision threshold with respect to each algorithm as described in Section 2.1.2.

The obtained results show that for Bozorth3, the probability value of a successful attack is equal to 0.0141% with the brute force attack and 0.0162% when we know the number of minutiae in the biometric reference. For the MCC algorithm, the probability value of a successful attack is equal to $1.63\times 10^{-4}$% considering the *brute force* attack and $1.6\times 10^{-4}$% by knowing the number of minutiae. We can see in both cases that the attacker gets a little gain with only this information.

In order to analyze whether the knowledge of the number of minutiae of the biometric reference has an impact on the effectiveness of this attack, we apply the following scenario: we only consider the scores between the reference template and the tests having the same number of minutiae. In this case, we have two sets of 4×800=3200 matching scores. We can calculate the $FAR_{A}$ value for both classes with the same number of minutiae. If we consider the Bozorth3 matching algorithm, the attacks succeed more for 1<ϵ<35 when the number of minutiae is greater than 38. For the MCC matching algorithm, the same remark can be formulated for 0.0011<ϵ<0.0023. Table 3 gives the probability value of a successful attack $FAR_{A}$ for each class of the number of minutiae for the two matching algorithms. We can see clearly that if we have more than 38 minutiae, this information helps the attacker more but it is not enough to significantly increase the success of the attack.

#### 2.2.3. Image Resolution

In terms of knowledge of the image resolution, we calculate the $FAR_{A}$ value for both scenarios when we set the decision threshold to get the value at the EER. The obtained results show that when we use the Bozorth3 matching algorithm, the probability value of a successful attack is equal to 0.019% with the brute force attack and 0.035% knowing the resolution of the original image. Considering the MCC algorithm, the probability value of a successful attack is equal to 0.51 $\times \;10^{-3}$% with the *brute force* attack and $0.8\times 10^{-3}$% knowing the resolution of the original image.

**Figure 5:**
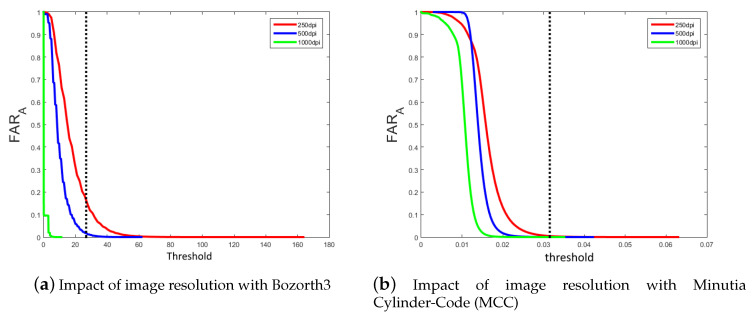
Evolution of the effectiveness of attacks by considering the three resolutions of the sensor for the two matching algorithms. The dot line corresponds to the threshold value associated to the Equal Error Rate (EER) performance (see Section 2.1.2).

We can see, in both cases, the small advantage for an attacker to know the resolution of the original image extracted by the sensor. In order to analyze whether the resolution of the original image has an impact on the efficiency of this attack, we apply the following protocol: we only consider the scores between the reference and attack models with the same resolution images. In this case, we have three sets of 4×800=3200 matching scores. Thus, we can compute the evolution of the $FAR_{A}$ value for each image resolution, as shown in Figure 5.

For the Bozorth3 matching algorithm, we can see that it is quite impossible to have a successful attack with a high resolution image (1000 dpi), as opposed to a low resolution image (250 dpi). The same remark can be formulated for the MCC algorithm. Table 4 gives the probability value of a successful attack $FAR_{A}$ for each image resolution for the two matching algorithms. We can clearly see that low resolution helps an attacker three times more than the average resolution (500 dpi). Avoiding the use of low-resolution images permits to limit this type of attack.

#### 2.2.4. Fingerprint Class

We also compute the value $FAR_{A}$ when the impostor knows the fingerprint class of the legitimate user to impersonate. Considering the Bozorth3 matching algorithm, the probability value of a successful attack is equal to 3% with the brute force attack and to 4.7% knowing the fingerprint class. The obtained results show that when using the MCC matching algorithm, the probability of a successful attack is equal to 1.7% with the brute force attack and 2.6% with the knowledge of the fingerprint class. We can deduce that the knowledge of the fingerprint class enrolled in the secure element helps an attacker to be authenticated on the system. However, we must study how this knowledge influences the effectiveness of the attack.

**Figure 6:**
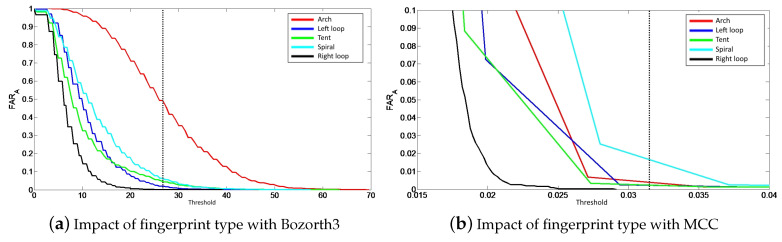
Evolution of attack efficiency taking into account all fingerprint classes for the two biometric systems.

In order to analyze its impact, we apply the following methodology: we only consider the scores between the reference template and attack probes having the same fingerprint class to calculate the $FAR_{A}$ value for each class. In this case, we have five series of 4×800=3200 matching scores allowing us to calculate the $FAR_{A}$ value. The results are presented in Figure 6. Considering the Bozorth3 matching algorithm, Figure 6a allows us to deduce that the Arch type has the highest success rate, whereas the right-loop has the lowest one. For the MCC matching algorithm, we observe in Figure 6b that the Whorl type has the highest attack rate contrary to the Right loop. A first remark that we can make is that the right loop fingerprints are the least simple to usurp. Table 5 gives the probability value of a successful attack $FAR_{A}$ for each fingerprint class for the two matching algorithms. We can clearly see that some fingerprint classes are easier to attack depending on the used matching algorithm. For example, with Bozorth3, Arch fingerprints can be spoofed in 50% of cases, which is very high.

### 2.3. Discussion

In this study, we wanted to know which a priori information could be important for an attacker in order to impersonate an individual enrolled on an embedded biometric system. We have shown that the knowledge that helps an attacker the most is the fingerprint class. Indeed, our experiments show that knowing the fingerprint class generally increases the probability of usurping a legitimate user. On the contrary, the knowledge of the number of minutiae, the sensor type or the image resolution are less informative. The algorithm on which we obtain the best rate of identity theft is Bozorth3 with Arch-type fingerprints. We hypothesize that this algorithm is less efficient and not optimized for Arch-type fingerprints. If we look at the other types of fingerprints for the two matching algorithms, we notice that the usurpation rate is quite low which is quite logical and coherent. Moreover, for both algorithms, the highest acceptance rate is on Whorl, which are the most common fingerprint type [19]. In general, we deduce that the success rate of an attack depends almost exclusively on the operation of the matching algorithm. We could extend this work by analyzing the combinations of a priori information.

In the next section, we propose a second contribution on fingerprint classification. This could be useful in detecting brute force and zero effort attacks, i.e., detecting attempts with different fingerprint classes.

## 3. Fingerprint Type Recognition

In the previous section, we identified many attacks consisting it sending a fake probe to the biometric system. The brute force attack is realized by sending random templates and the Zero effort by one some real samples captured directly from the impostor. Both attacks could be easily detected if the fingerprint class of the probe does not correspond to the legitimate one. Consequently, it is necessary to recognize the fingerprint class from an ISO fingerprint template [20]. Because the fingerprint image is not always available (not possible to store the fingerprint image in the SE), we consider in this work that we only have the minutiae template to achieve this goal. In the next section, we make a literature review on fingerprint classification.

### 3.1. State-of-the-Art Review

In 1996, Karu and Jain defined the first method for fingerprint classification based on singular points in fingerprint images [21]. They obtained very good results on the NIST SD4 dataset [22] (accuracy of 93% for 4 and 5 classes recognition). Li et al. [23] in 2008 proposed an algorithm based on the interactive validation of singular points and the constrained nonlinear orientation model. The final features used for classification are the coefficients of the orientation model and the singularity information. They obtained an accuracy of 95% on the NIST SD4. In 2013, Cao et al. [24] proposed a regularized orientation diffusion model for fingerprint orientation extraction combined with a hierarchical classifier for fingerprint classification. They obtained very good results on the NIST SD4 dataset, i.e., a classification accuracy of 95.9% for five-class classification and 97.2% for four-class classification without any rejection. In 2016, Wang et al. [25] proposed a deep learning approach for fingerprint classification. They obtained on the NIST SD4 dataset an accuracy of 91%.

All these methods provide very good results but require having the fingerprint image as input. This induces a lot of computational resources as well as time. This is not possible in embedded biometric systems. Indeed, it is not possible to store the fingerprint image in such devices, only the minutiae template is available mainly due to memory limitation. Our objective is to propose a fingerprint classification method with only the minutiae template as input, i.e., without any access to the fingerprint image. However, very few works have considered this problem. To the best of our knowledge, the paper by Ross et al. [26] is the only work addressing this problem. The proposed method uses minutiae triplet information to estimate the orientation map of the associated fingerprint. They obtained, on the NIST SD4 dataset, an accuracy of 82%.

### 3.2. Proposed Method

In this study, we only process ISO Compact Card II minutiae templates. This format consists of four features $\left(x_{i},y_{i},T_{i},\theta_{i}\right),\;i=1:N_{j}$ where:

- $\left(x_{i},y_{i}\right)$ corresponds to the location of the minutiae in the image (the image being of course unavailable),
- $T_{i}$ is the type of the minutiae (bifurcation or ridge ending),
- $\theta_{i}$ is the orientation of the minutiae relative to the ridge. This information is represented by 6 bits, i.e., it has 64 different values.
- $N_{jk}$ is the number of minutiae for the sample *j* of the user *k*.

The proposed method consists in defining new features from the minutiae template.

#### 3.2.1. Features Computation

From the minutiae template, we design a first statistical features vector called IsoStruct${}_{jk}$. For each parameter of this vector, the normalized histogram is generated with a fixed quantization level. We normalize the histograms in order to be invariant to the number of minutiae present in each template. We obtain then an IsoStruct${}_{jk}$ vector of size 3×NQ+2 by concatenating these histograms, where NQ is the number of quantization level in the histogram computation and the value 2 corresponds to the histogram built on the type of minutiae that contains only two different values. This statistical vector IsoStruct${}_{jk}$ is then defined as follows:

(2) $${\mathrm{IsoStruct}}_{jk}=\left\{{\mathrm{HistoX}}_{jk},{\mathrm{HistoY}}_{jk},{\mathrm{HistoIsoAngle}}_{jk},{\mathrm{HistoType}}_{jk}\right\},$$

where ${\mathrm{HistoX}}_{jk},{\mathrm{HistoY}}_{jk},{\mathrm{HistoIsoAngle}}_{jk}$ and ${\mathrm{HistoType}}_{jk}$ are normalized histograms. In order to have several levels of precision on each of the histograms, they are generated with a variable number NQ of quantification levels.

**Figure 7:**
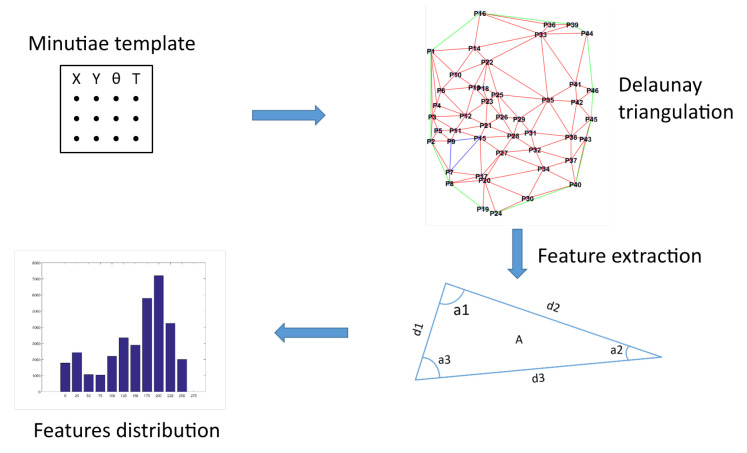
Features computation from the Delaunay triangulation (source [20]).

**Figure 8:**
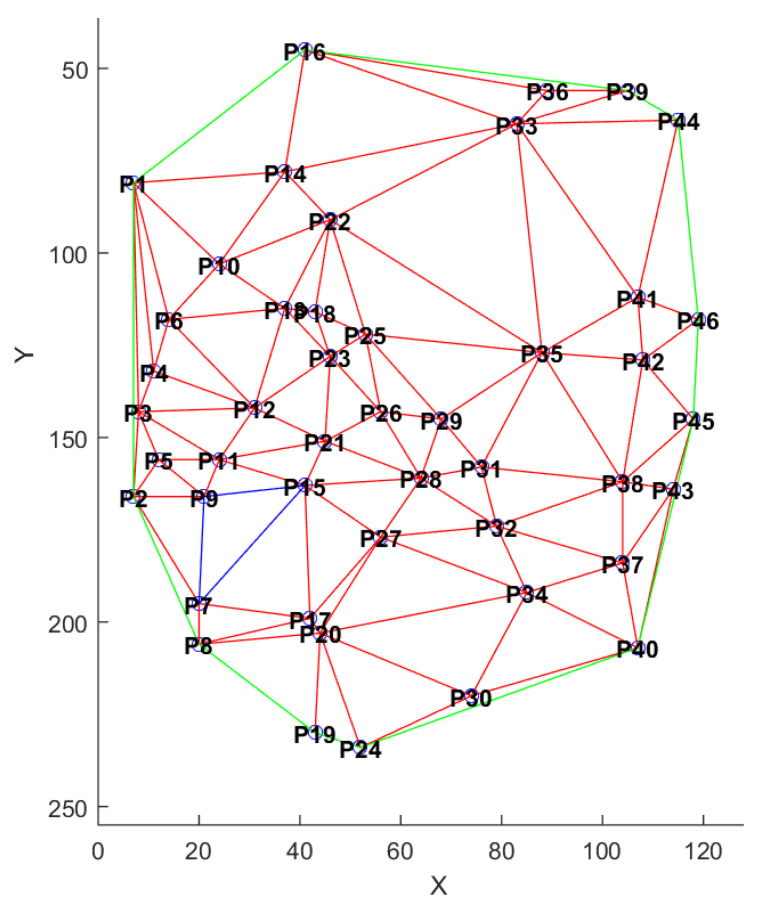
Example of Delaunay triangulation of a minutiae template (source [20]).

In order to take into account the spatial distribution of minutiae, we propose to use the Delaunay triangulation as translation and rotation invariant representation [27,28]. Delaunay triangulation is used in various domains, such as algorithmic geometry [29] to solve problems, or in surface reconstruction [30,31,32]. This representation has often been used for digital fingerprints [33,34,35]. This representation allows us to create a structure containing parameters describing each template, as shown in Figure 7. This structure is composed of many elements: length of the edges of the triangles, the angles, the area of the triangles and thei perimeter. Figure 8 shows an example of triangulation obtained by considering the minutiae as the vertices of the generated triangles. For each triangle, we extract (1) all the three angles values, (2) all the three edge lengths, and (3) the triangle area.

Thus, each template *j* of an individual *k* can be represented by a feature vector ${\mathrm{TriInf}}_{jk}$ composed of three sets of parameters:

(3) $$\begin{array}{c} {\mathrm{TriInf}}_{j,k}=\{\left\{{\mathrm{AngleA}}_{jkl},{\mathrm{AngleB}}_{jkl},{\mathrm{AngleC}}_{jkl}\right\},\\ \;\{{\mathrm{LengthAB}}_{jkl},{\mathrm{LengthAC}}_{jkl},{\mathrm{LengthBC}}_{jkl}\},\\ \;\{{\mathrm{Area}}_{jkl}\}\},\forall l\in \left[1;M_{jk}\right], \end{array}$$

where $\left\{{\mathrm{AngleA}}_{jkl},{\mathrm{AngleB}}_{jkl},{\mathrm{AngleC}}_{jkl}\right\}$ is the vector of angle values of the $M_{jk}$ triangles of the template *j* for user *k*. $\left\{{\mathrm{LengthAB}}_{jkl},{\mathrm{LengthAC}}_{jkl},{\mathrm{LengthBC}}_{jkl}\right\}$ represents the vector of lengths for each triangle and $\left\{{\mathrm{Area}}_{jkl}\right\}$ is the data vector associated to the area of the triangles. We added some parameters concerning the orientation of minutiae even if this parameter is not related to the Delaunay triangulation:

(4) $$\begin{array}{c} {\mathrm{IsoAngleInf}}_{j,k}=\left\{{\mathrm{Orientation}}_{jki}\right\},\forall i\in \left[1;N_{jk}\right], \end{array}$$

where ${\mathrm{Orientation}}_{jki}$ represents the vector of angles of the $N_{jk}$ minutiae in the template *j* of user *k*. From these two feature vectors ${\mathrm{TriInf}}_{jk}$ and ${\mathrm{IsoAngleInfo}}_{jk}$, a new statistical vector is generated. We compute a normalized histogram to approximate a probability density for each characteristic that is not dependent on the number of minutiae in the template. These histograms are calculated by considering a fixed value of the quantization level. We then obtain a TemplateStruct${}_{jk}$ vector of size 4×NQ, where NQ is the number of quantization levels in the histogram calculation. This statistical vector TemplateStruct${}_{jk}$ is obtained by a histogram concatenation defined as follows:

(5) $$\begin{array}{c} {\mathrm{TemplateStruct}}_{jk}=\{{\mathrm{HistoAngle}}_{jk},\\ \;{\mathrm{HistoDistance}}_{jk},{\mathrm{HistoArea}}_{jk},\\ \;{\mathrm{HistoISOAngle}}_{jk}\}, \end{array}$$

where ${\mathrm{HistoAngle}}_{jk},{\mathrm{HistoDistance}}_{jk},{\mathrm{HistoArea}}_{jk}$ et ${\mathrm{HistoISOAngle}}_{jk}$ are normalized histograms calculated from their associated subvector TriInf${}_{jk}$ and IsoAngleInfo${}_{jk}$. These histograms are generated with a variable number of levels NQ of quantization, allowing to refine the precision of the histogram.

#### 3.2.2. Machine Learning

In order to define a model for each of the five classes of fingerprints, we use a statistical learning technique. From all the existing classification schemes, we have chosen the technique based on Support Vector Machine (SVM) because of its high classification rate obtained in many works [36,37,38], as well as its high level of generalization. We tested other machine learning algorithms (such as Naive Bayes or Adaboost) but SVM provided the best results. SVMs have been developed by Vapnik et al. [39] and are based on the principle of minimizing the structural risks of statistical learning theory. SVMs express predictions in terms of linear combination of kernel functions centered on a subset of the learning data, called support vector (SV).

Since SVMs are binary classifiers, several binary SVM classifiers are required for a multi-class classification problem when using a SVM classification technique. A final decision is then taken from all the outputs of the [37] binary SVMs. The choice of the function of the kernel is essential. The RBF (Radial Basis Function) kernel function is commonly used with SVM. The main reason is that the RBF functions can be considered as similarity measures between two examples. A final decision must be made from all binary decision functions. Several combining strategies can be used [37]. Among all the existing strategies, majority voting was chosen in our study for its simplicity of implementation.

### 3.3. Experimental Protocol

We enumerate here all the elements necessary for our experiments.

SFinge databases: The FVC databases usually used in fingerprint works do not provide any information about the fingerprint class. We have therefore generated five databases with the SFinge software [11], one for each fingerprint class as described in Table 6. Each generated database contains 800 biometric samples.

In order to recognize the fingerprint class associated with a template, it is necessary to train the SVM on a learning dataset. A test dataset is required to measure the effectiveness of the generated classifier. To do this, several test-learning sequences have been executed. In each sequence, the fingerprint database was subdivided into separate sets for learning and tests. In each test, 80% of the database was selected for learning and 20% for testing for each of the 5 databases. More specifically, each training set contains 640 fingerprints, while the test set contains the remaining 260 fingerprints. We performed 1000 random draws have and the recognition rate is averaged. We used the libsvm library [40] with the default settings. The minutiae templates used in the experiments have been extracted using the NBIS tool, and more specifically MINDTCT [41] from NIST.

### 3.4. Experimental Results

We analyze the efficiency of the two proposed features vectors (IsoStruct and TemplateStruct) in the two next sections.

#### Isostruct Features

One of the first elements we need to test is the number of quantization levels in the normalized histograms. We tested different quantization levels (8, 16, 32, 64) on the structure of the characteristics. The results are presented in Table 7. We can observe that the best results are obtained with 16 quantization levels for the structure based on the characteristics. For a quantization level equal to 64, one observes a severe decrease of the recognition rate (60.8%). This can be explained by the fact that at this quantization level, redundancy is introduced since many zero values are generated. Considering a feature vector composed of 50 values (50=3×16+2), we obtain 80.37% as recognition rate of the fingerprint class with SVM (without any optimization). In the following, we keep this vector feature size.

Table 8 presents the obtained results for each fingerprint class. We find that the most recognized fingerprint classes are Spiral, Arch and Tented with respectively with accuracy 87%, 85% and 80%. We notice that left and right loops have a recognition rate of 75% which is low compared to the other classes. We have noticed that loops (whether left or right), are often confused by the SVM, which explains this result.

Since the minutiae template contains only four pieces of information, we want to know which ones are important for the fingerprint classification. Table 9 indicates the recognition rate for each quantization level value and for each parameter in the minutiae template. We can observe that the H(Type) has the same recognition rate regardless of the number of used quantization levels. This is due to the two possible values of this parameter, we only have a histogram with two quantization levels compared to the other parameters. As for the histograms on the H(X) and H(Y) minutiae position, we have poor results with about 40% recognition rate. This was predictable because the position of the minutiae depends on the interaction between the finger and the sensor. A finger can thus be placed sideways on the latter and strongly impacts the recognition of the fingerprint type. On the contrary, with the histogram of angles, H(ISO_Angle), we have the best recognition rate of the fingerprint type. These results are coherent because angles are calculated from the ridges of the fingerprint and the orthonormal coordinates of the sensor, which gives a general idea of the direction of the various minutiae and thus finds the fingerprint type easier.

We can conclude that the characteristic H(ISO_Angle) is an important information for the recognition of the fingerprint class. With about 80% recognition rate, this is the most important parameter present in the initial template, the other three parameters do not improve performance. These results are satisfactory, but we wish to have more relevant information and thus improve the efficiency.

### 3.5. Templatestruct Features

We used the same protocol as defined in the Section 3.3 and the number of quantization levels defined in the Section 3.4 with N= 16. Table 10 gives the accuracy for the TemplateStuct features vector. If we compare the results with the IsoStruct features vector, we have a great difference of about 9% for the accuracy. This feature obtains a recognition rate of 89%.

When performing the same procedure as before, we obtain the correct classification rates presented in Table 11 for each fingerprint class. We note once more that the Spiral and Arch fingerprint classes are better recognized with an accuracy more than 95%. These results are very satisfactory because it shows that the TemplateStruct features provide more information and allow the SVM to better categorize fingerprints. The Tented class is recognized at 89% with an improvement of 9% compared to the result presented previously. Once again, left and right loops have the lowest accuracy with 82% and the SVM despite the addition of information with our method always has difficulty to differentiate these two classes. These features could certainly be improved, and other considerations could be taken into account like smaller or larger angles in the Delaunay triangulation.

### 3.6. Discussion

The addressed problem in this paper is to determine the fingerprint class given the minutiae template. We proposed two methods mainly based on the proposal of features vectors. The first one concatenates histograms of each information in the minutiae template. With this approach (called IsoStruc), we get 80.37% as the recognition rate. We observed that the orientation of minutiae was the most discriminating parameter allowing to achieve a good recognition rate of 80.23%. This is why the second feature vector is based on a geometric approach based on the Delaunay triangulation, allowing us to obtain more parameters while keeping the ISO_Angle parameter. With this method, we increase the recognition rate by 9% and we get 89% of accuracy for fingerprint classification.

Table 12 presents the accuracy of proposed methods in the literature for fingerprint classification. We can see that the proposed methods (especially the one based on the TemplateStruct features vector) provides very good results. When only the minutiae template is available, we obtain the best results that are not far from methods processing fingerprint image.

## 4. Conclusions and Perspectives

We have demonstrated that only two a priori information help acceptance by the system, the image resolution and, most significantly, the fingerprint type. Perspectives of this work concern the study of other a priori information that could be interesting for an attacker. We could think of the knowledge of the used sensor (brand, specifications) for the enrollment or gender as for example. A combination of studied a priori information could be investigated to confirm these results.

We proposed in this study some new features allowing us to reach an accuracy of 89% for fingerprint classification, given a minutiae template as input. In order to improve the fingerprint classification, we could think of using deep learning approaches to increase the accuracy.

## Figures and Tables

**Table 1:** Example of scenario 2 for the sensor type.

Reference BDD	Attack BDD
Capacitive	Capacitive
Capacitive	Optical
Optical	Capacitive
Optical	Optical

**Table 2:** Probability value of a successful attack $FAR_{A}$ for each sensor type for the two matching algorithms.

Matching Algorithm	Capacitive	Optical
Bozorth3	0.0158%	0.016%
MCC	$0.13\times 10^{-3}$%	$0.23\times 10^{-3}$%

**Table 3:** Probability value of a successful attack $FAR_{A}$ for the two classes of the number of minutiae for the two matching algorithms.

Matching Algorithm	<38	>38
Bozorth3	0.0038%	0.0391%
Minutia CC	$0.8\times 10^{-4}$%	$2.5\times 10^{-4}$%

**Table 4:** Probability value of a successful attack $FAR_{A}$ for each resolution of the original image for the two matching algorithms.

Matching Algorithm	250 dpi	500 dpi	1000 dpi
Bozorth3	0.165%	0.047%	0%
Minutia CC	$0.45\times 10^{-3}$%	$0.176\times 10^{-3}$%	0%

**Table 5:** Probability value of a successful attack $FAR_{A}$ for each fingerprint class for the two matching algorithms.

Matching Algorithm	Arch	Right Loop	Left Loop	Tented	Whorl
Bozorth3	50%	0%	2%	5%	6.3%
Minutia CC	0.6%	0%	0.2%	0.2%	2%

**Table 6:** Label for generated databases for each fingerprint class.

Label	Fingerprint Type
1	Arch
2	Left loop
3	Right loop
4	Tented
5	Whorl

**Table 7:** Fingerprint classification results with the IsoStruct features vector.

Quantization Levels	Recognition Rate (%)—IsoStruct
8	79.43
16	80.37
32	80.06
64	60.80

**Table 8:** Fingerprint classification results with the IsoStruct features vector.

	Arch	Left Loop	Right Loop	Tented	Whorl
Recognition rate—IsoStruct (%)	85	75	75	80	87

**Table 9:** Fingerprint classification results for each component of the IsoStruct features vector.

	Recognition Rate—IsoStruct (%)			
**Quantification Level**	***H(X)***	***H(Y)***	***H(ISO_Angle)***	***H(Type)***
8	42.87	37.52	77.85	28.13
16	43.62	38.96	80.23	28.13
32	42.25	36.51	80.24	28.13
64	40.45	36.47	78.25	28.13

**Table 10:** Comparison of fingerprint classification results for the 2 proposed features vectors.

	Recognition Rate (%)
IsoStruct	80.37
TemplateStruct	89.12

**Table 11:** Fingerprint classification results with the TemplateStruct features vector.

	Arch	Left Loop	Right Loop	Tented	Whorl
Recognition rate (%)	95	82	82	89	97.8

**Table 12:** Comparison with methods from the state of the art.

Methods	Input	Accuracy
Karu and Jain [21]	image	93%
Li et al. [23]	image	95%
Cao et al. [24]	image	96%
Wang et al. [25]	image	91%
Ross et al. [26]	minutiae	82%
Proposed (IsoStruct)	minutiae	80%
Proposed (TemplateStruct)	minutiae	89%
